# Neighborhood Socioeconomic Deprivation and Allostatic Load: A Scoping Review

**DOI:** 10.3390/ijerph15061092

**Published:** 2018-05-28

**Authors:** Ana Isabel Ribeiro, Joana Amaro, Cosima Lisi, Silvia Fraga

**Affiliations:** 1 EPIUnit–Instituto de Saúde Pública, Universidade do Porto, 4050-600 Porto, Portugal; joana.amaro@ispup.up.pt (J.A.); clisi@ispup.up.pt (C.S.); silfraga@med.up.pt (S.F.); 2 Departamento de Ciências da Saúde Pública e Forenses e Educação Médica, Faculdade de Medicina, Universidade do Porto, 4200-319 Porto, Portugal

**Keywords:** socioeconomic factors, cumulative biological risk, health disparities, neighborhood effects, allostatic load, neighborhood disadvantage, poverty, context, biomarkers, residence characteristics

## Abstract

Residing in socioeconomically deprived neighborhoods may pose substantial physiological stress, which can then lead to higher allostatic load (AL), a marker of biological wear and tear that precedes disease. The aim of the present study was to map the current evidence about the relationship between neighborhood socioeconomic deprivation and AL. A scoping review approach was chosen to provide an overview of the type, quantity, and extent of research available. The review was conducted using three bibliographic databases (PubMed, SCOPUS, and Web of Science) and a standardized protocol. Fourteen studies were identified. Studies were predominantly from the USA, cross-sectional, focused on adults, and involved different races and ethnic groups. A wide range of measures of AL were identified: the mode of the number of biomarkers per study was eight but with large variability (range: 6–24). Most studies (*n* = 12) reported a significant association between neighborhood deprivation and AL. Behaviors and environmental stressors seem to mediate this relationship and associations appear more pronounced among Blacks, men, and individuals with poor social support. Such conclusions have important public health implications as they enforce the idea that neighborhood environment should be improved to prevent physiological dysregulation and consequent chronic diseases.

## 1. Introduction

Residing in deprived neighborhoods, characterized by a concentration of impoverished, less educated people and by poor living conditions, has been associated with increased risk of disease [1] and death [2,3] even after accounting for individual-level characteristics. Several mechanisms may explain this “miasmatic” health effect of neighborhood socioeconomic deprivation. Wealthy neighborhoods tend to attract beneficial facilities, such as healthy food shops [4], cultural and recreational places [5], and keep away toxic and harmful exposures such as air pollution [6], waste dumps or industries [7], which are often disproportionally concentrated in disadvantaged areas. Furthermore, the socioeconomic structure of neighborhoods also influences behaviors, aspirations and social norms shared by residents [8]. For instance, unhealthy coping behaviors such as alcohol consumption and smoking are more common in disadvantaged neighborhoods, not only due to the presence of infrastructures (tobacco/alcohol retailers) that potentiate such behaviors, but also because these behaviors are more widely accepted by the community [9,10]. Hence, according to the “deprivation amplification model” [11], residents in poor neighborhoods are not only personally poor, but they are also more likely to reside in neighborhoods that lack the opportunities to lead a healthy life [12].

Most of the studies about the influence of neighborhood socioeconomic deprivation on health have focused on downstream health outcomes—onset of death or disease [1,2]—and little research has been conducted to investigate the biological consequences and pathways that link the exposure to harmful environmental factors to disease pathogenesis [13], including elevated inflammation [14], greater responses to stress [15], or higher allostatic load [16].

Firstly introduced in the 1990s by McEwen and Stellar [17], the allostatic load, AL, sometimes labeled cumulative biological risk, is a theoretical construct that represents dysregulation across the body’s multiple physiological systems responsible for maintaining equilibrium when faced with physical or social challenges [18,19]. Physiological responses are initiated as the body tries to achieve stability (allostasis) after being exposed to physical and social stressors [16]. When these exposures become chronic and repeated, physiological responses to stress can accumulate and lead to an overexposure to neural, endocrine, and immune stress mediators, which in turn can lead to permanent negative health outcomes in different peripheral biological systems, such as the cardiovascular and metabolic systems [16].

Behind AL is the view that biological systems work both additively and synergistically to influence disease pathogenesis. For that reason, the AL is operationalized as a combination of biomarkers including primary mediators (stress-related hormones that help maintain homeostasis after exposure to stressful stimuli, e.g., cortisol) and secondary outcomes (sub-clinical disturbances in markers of cardiovascular, metabolic, and immune functioning, e.g., blood lipids) [18,19]. The original version of the AL index included six secondary outcomes—systolic and diastolic blood pressure, total cholesterol, high-density-lipoprotein (HDL), glycosylated hemoglobin (HbA_1C_), waist-to-hip ratio—and four primary mediators—dehydroepiandrosterone sulfate, urinary epinephrine, norepinephrine, and cortisol [18]. The AL index itself is derived by summing the number of biomarkers for which the subject fell into the highest risk quartile [18]. However, many subsequent studies have used different combinations of biomarkers [19]. Despite the wide variation in the way AL is measured, results are consistent: higher AL has been associated with an increased risk of cardiovascular disease, cognitive decline, physical limitations, and all-cause mortality [18,20,21]. Indeed, the aggregate AL index can predict morbidity and mortality risks better than its individual components [22].

Residing in disadvantaged areas may pose substantial physiological stress, as people living in those places are more likely to engage in unhealthier behaviors and to be exposed to social and environmental stressors [20]. With this in mind, a recent body of literature has evaluated the association between neighborhood socioeconomic deprivation and AL [20]. Despite this is an emergent and promising research topic, no systematic review was conducted to systematically summarize the published evidence and to discuss the main methodological and conceptual challenges underpinning this emerging research line. To understand how unhealthy environments are embedded to create health disorders, we conducted a scoping review of published evidence to assess what is known about the relation between neighborhood socioeconomic deprivation and AL.

## 2. Materials and Methods

Our scoping review was guided by the five stage framework proposed by Arksey and O’Malley [23]—research question; relevant studies; selection of studies; charting data; and collating, summarizing, and reporting results—and subsequent recommendations [24] to ensure compliance with current standards for conducting a scoping review.

*Stage 1: Research question*

To guide the present review, we focused on the following main research question: “What is known about the association between AL and neighborhood socioeconomic deprivation?”.

*Stage 2: Relevant Studies*

We conducted a scoping review on AL and neighborhood socioeconomic deprivation by searching PubMed, SCOPUS, and Web of Science from 3 January to the 1 March 2018. We used the following set of key-words—allostatic, allostasis, cumulative biological risk, cumulative physiological risk, cumulative stress, deprivation, socioeconomic, poverty, disadvantaged—combined with the study scales—area, place, neighborhood, neighborhood, state, county, counties, region, residence, residential. Then, all references were imported into a reference management software (i.e., Endnote X4, Clarivate Analytics (Philadelphia, PA, USA)).

*Stage 3: Selection of Studies*

**Figure 1:**
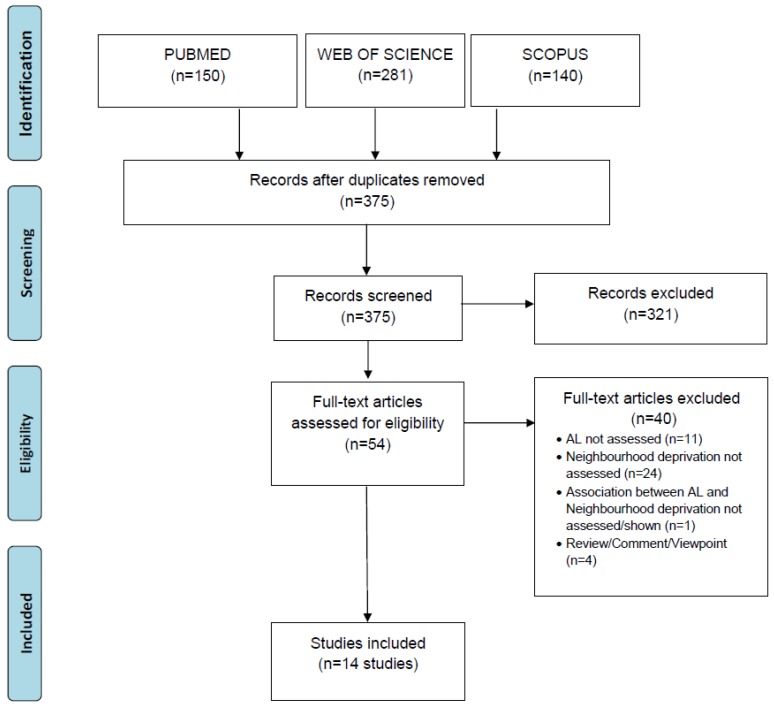
PRISMA diagram of the study selection process.

We included all studies conducted in humans that assessed the association between neighborhood socioeconomic deprivation and AL. The following exclusion criteria were applied: 1-non-human studies; 2-study type (reviews, reports, editorials, comments); 3-neighbourhood socioeconomic deprivation not assessed; 4-AL not assessed; and 5-association between neighborhood deprivation and AL not assessed. No temporal, geographical or language restrictions were imposed. Once all references were gathered in the reference database, duplicates were eliminated (*n* = 196) leaving us with a database of 375 studies. Then, two reviewers screened the titles and abstracts to identify studies that did not meet the inclusion criteria or without full-text available (*n* = 321: 319 did not met the inclusion criteria and from two full-text was not available). Afterwards, full-texts of potentially eligible studies were read and those that did not meet the inclusion criteria were eliminated (*n* = 40). In case of divergent opinions between the two reviewers, consensus was reached by a third reviewer. New potentially relevant studies were sought by forward and backward citation tracking of the articles included in the review; no additional papers were found. The PRISMA (Preferred Reporting Items for Systematic Reviews and Meta-Analyses) [25] flowchart from Figure 1 depicts the study selection process.

*Stage 4: Charting Data*

Using a standardized data collection, we gathered key pieces of information from the selected articles: author, year of publication, country, study design, aim, sample characteristics (size, study/cohort name, region, participant’s age, gender and ethnicity), methods (how measures of AL and neighborhood socioeconomic deprivation were derived, control for confounding, studied mediators and moderators), and key findings. 

*Stage 5: Collating, Summarizing, and Reporting Results*

We synthesized the data according to the previously mentioned research question and scope of inquiry. Tables and figures were created to systematize and summarize the information.

## 3. Results

### 3.1. General Characteristics

The literature search yielded 14 eligible papers [10,16,26,27,28,29,30,31,32,33,34,35,36,37], published from 2009 to 2016. Detailed information on their main characteristics can be found in the Supplementary Table 1 (Table S1). Most studies were conducted in the USA, except for one study that was conducted in Sweden [32]. The mean sample size was 3068 participants, ranging between 420 [31] and 13,199 [26]. Twelve out of the 14 studies used adult samples [16,26,27,28,29,30,32,34,35,36,37,38] and two focused on youth [31,33]. Seven studies included participants of different races/ethnicities [16,26,27,28,29,30,38], four studies exclusively focused on Blacks [31,33,35,36], and one on Hispanics [34]. The majority of the studies relied on a cross-sectional design (*n* = 10) [16,26,27,28,29,30,35,36,37,38], whereas four studies were longitudinal [31,32,33,34].

### 3.2. Measurement of Allostatic Load

**Figure 2:**
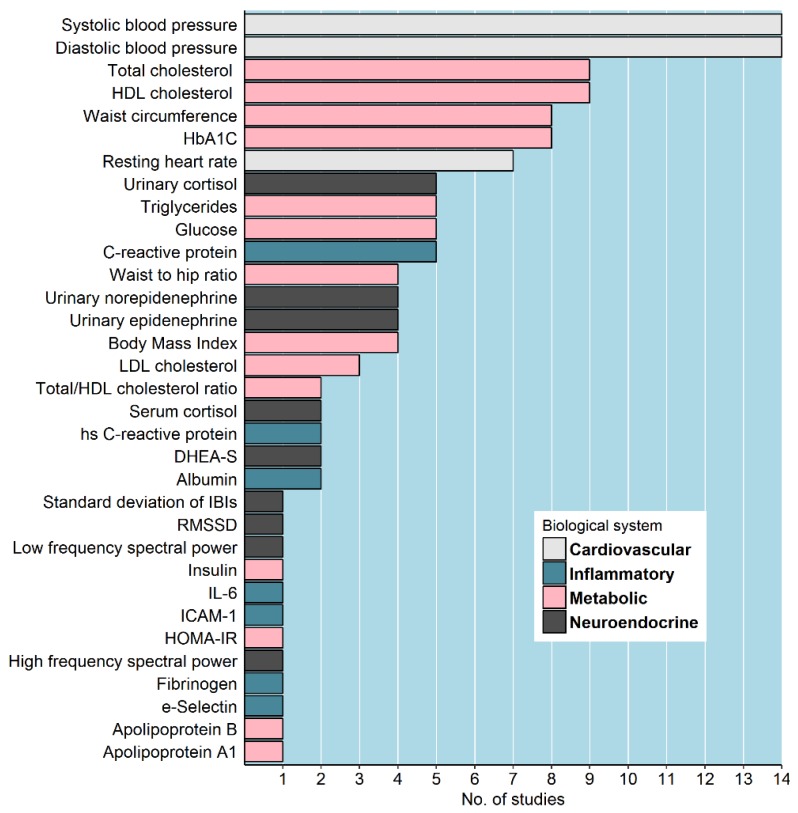
Biomarkers used in the included studies according to biological system. DHEA-S = Dehydroepiandrosterone sulfate; HbA1C = glycosylated hemoglobin; HDL = High-density lipoprotein; HOMA-IR = Homeostatic model assessment insulin resistance; IBIs = Inter-beat intervals; ICAM-1 = Intercellular adhesion molecule 1; IL-6 = Interleukin 6; LDL = Low-density lipoprotein; RMSSD = Root Mean Square of the Successive Differences (Heart rate variability).

As depicted in Figure 2, a wide range of measures of AL was used in the included studies. The mode of the number of biomarkers per study was eight but with large variability—from 24 [37] biomarkers to six biomarkers [31,33]. All the studies included secondary outcomes to measure AL, whereas only seven used primary mediators [31,32,33,34,35,36,37]. The most commonly used primary mediators were urinary cortisol (*n* = 5) [31,32,33,34,37], epinephrine (*n* = 4) [31,33,34,37], and norepinephrine (*n* = 4) [31,33,34,37]. Among the studies that included cortisol measurements (*n* = 7) [31,32,33,34,35,36,37], three accounted for circadian fluctuations [31,32,33]. The most widely used secondary outcomes were the markers related with cardiovascular system dysregulation (systolic and diastolic blood pressure), which were used in all the included studies [16,26,27,28,29,30,31,32,33,34,35,36,37,38], followed by total and high density cholesterol, which were utilized in nine studies [16,26,27,28,29,30,32,34,38] to derive the AL index. Additionally, in more than half of the studies, waist circumference (*n* = 8) [16,28,29,30,32,35,36,38] and HbA_1C_ (*n* = 8) [26,27,28,34,35,37] were used. Regarding inflammatory markers, C-reactive protein was, by far, the most widely used biomarker [26,27,28,31,32,38]. Large differences were also seen in the way the AL index was calculated. About half of the studies have used clinically defined criteria for high risk [16,26,27,28,29,34,38], three have categorized biomarker’s values according to quartiles [30,31,37], one according to tertiles [32], and three studies used the full spectrum of values by summing up biomarker’s z-scores [33,35,36]. Only five studies clearly state they have corrected the AL index for the lowering effect of medication use [16,29,34,35,38]. Besides the overall AL index, sub-indexes according to biological system were used in three of the included studies [27,35,36].

### 3.3. Definition of Neighbourhood and Measurement of Neighbourhood Socioeconomic Deprivation

**Figure 3:**
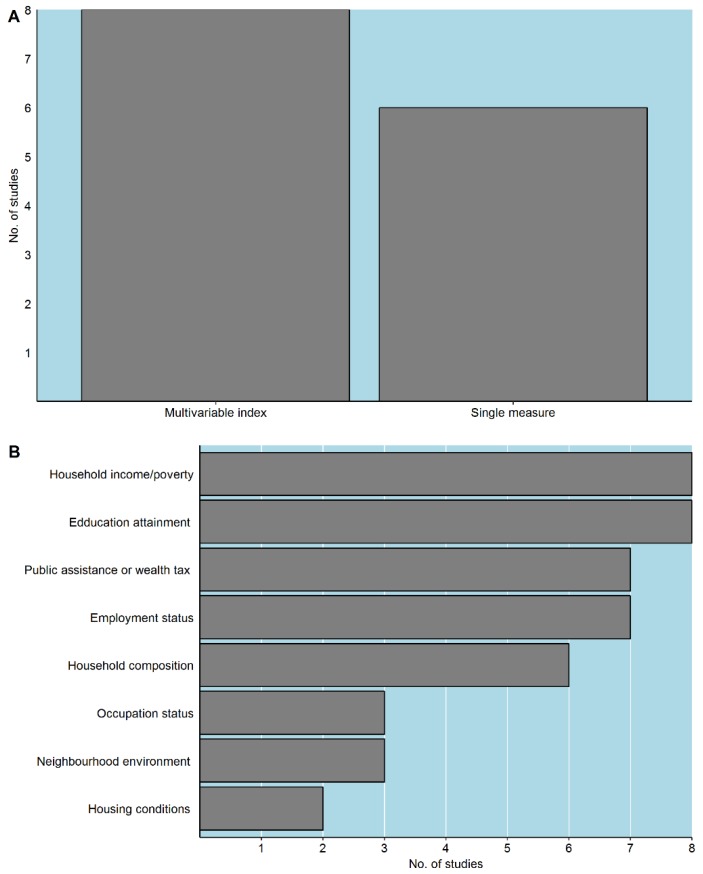
Measures of neighborhood socioeconomic deprivation used in the included studies: (**A**) No. of studies using single measures vs. multivariable indexes of neighborhood socioeconomic deprivation; (**B**) Variables included in the multivariable indexes of neighborhood socioeconomic deprivation.

As shown in Figure 3, in most of the studies (*n* = 8) [26,27,28,32,34,35,36,38], neighborhood socioeconomic deprivation was assessed using multivariable indexes of affluence and/or deprivation covering multiple domains—education (*n* = 8) [26,27,28,32,34,35,36,38]; income/poverty (*n* = 8) [26,27,28,32,34,35,36,38]; public assistance or wealth tax payment (*n* = 7) [26,27,28,32,34,35,36]; employment status (*n* = 7) [26,27,28,32,34,35,36]; household composition (*n* = 6) [26,27,32,34,35,36]; among others. Variables related with housing conditions (*n* = 2) [35,36] and neighborhood environment (more precisely, the presence of vacant housing units) (*n* = 3) [28,35,36] were less often included. Single measures of neighborhood socioeconomic deprivation were also frequently utilized: five studies relied on the proportion of households below the poverty line [16,29,30,31,33] and one on the household mean income [37]. Area-based socioeconomic characteristics were computed at different scales and levels of aggregation. The Swedish study [32] used Small-Area Market Statistics, composed of approximately 1000 residents, whereas the US studies aggregated participants either within census tracts (1200–8000 inhabitants) [26,27,28,31,33,35,36,37,38], or within block groups (600–3000 inhabitants) [16,29,30,34]. Regarding the geographic units and the definition of neighborhood, all the 14 studies used administrative divisions as proxies of residential neighborhood.

### 3.4. Associations

Table 1 summarizes the main findings obtained in the included studies. Most reported a significant and positive association between neighborhood deprivation and AL (*n* = 12) even after controlling for confounding variables [16,26,27,28,29,31,32,33,35,36,37,38], meaning that individuals residing in more deprived neighborhoods presented higher AL. Some studies (*n* = 3) tested the possibility that neighborhood deprivation would have a higher toll on certain biological systems. In these system-specific analyses, Barber et al. found stronger associations with the sub-indexes of the metabolic and neuroendocrine system [35,36]. Bird et al. also found a stronger influence over the metabolic system, but they also observed that the sub-index related with cardiovascular dysregulation was one of the most affected biological systems [27].

### 3.5. Confounding and Robustness Checks

Control for confounding was present in all the investigations, as most studies adjusted their results for individual socioeconomic status, gender, age, ethnicity as well as health-related behaviors. Yet, it is important to mention that only four studies accounted for residential mobility, i.e., residential trajectories and/or the amount of life time participants spent in each neighborhood [16,31,32,34]. Finally, only a minority of studies (*n* = 2) ran a sensitivity analysis to test whether the methods to compute the AL index were driving their results: Merkin et al. examined the associations using AL index both as a continuous and a count variable [26] and King and co-authors examined the robustness of the results to alternate specifications of the index by serially excluding each item and recalculating the index [28].

### 3.6. Moderators

Moderators are variables that modify the measure of effect of a putative causal factor under study [39]. More than half of these studies (*n* = 8) posited that the association between AL and neighborhood deprivation would differ according to socioeconomic and demographic strata: race/ethnicity, social support, individual socioeconomic status (SES), and gender. Indeed, several studies confirmed this premise (*n* = 5) [26,31,32,33,36]. Significant associations between neighborhood socioeconomic deprivation and AL were observed in men but not in women [32,36] and in Blacks but not in Whites or Hispanics [26] Social support also modified the association between neighborhood deprivation and AL, which was only evident among individuals that did not received high levels of emotional support [31]. Similarly, neighborhood social cohesion marginally modified the effect of neighborhood disadvantage—one of the included studies found that the association between neighborhood deprivation and AL is larger among individuals residing in neighborhoods with low levels of social cohesion [36]. Finally, testing the skin-deep resilience hypothesis (i.e., if the effort that deprived individuals must mobilize to succeed lead to higher AL), Chen and co-authors found that youth from deprived neighborhoods that went to college presented higher AL, but no differences were observed among those that did not [33]. On the other hand, it is important to refer that some of the included studies found no differential effects by race [16,27], gender [27], age [29] and individual socioeconomic status [27,29].

### 3.7. Mediators

Mediators are variables that occur in a causal pathway from a causal variable to an outcome variable [39]. To reach a deeper understanding of the underlying mechanisms, three studies investigated the potential pathways that connect neighborhood socioeconomic deprivation and AL [16,29,37]. Robinette and colleagues, examined whether lower neighborhood income would relate to higher AL, through psychological (i.e., neighborhood safety and cohesion), affective (anxiety, stress), and behavioral pathways (poor diet, physical inactivity and smoking) and found that the associations between AL and neighborhood deprivation were indeed partially explained by affective and behavioral pathways, but not by psychological pathways [37]. Contrastingly, Schulz et al. in 2012 concluded that the relationships between neighborhood deprivation and AL were mediated by self-reported neighborhood environment stressors (e.g., unsafe feelings, unfair treatment, physical environmental stressors), but not by health behaviors [16]. In 2013, Schulz and co-authors evaluated the extent to which the association between neighborhood deprivation and AL was explained by objective and perceived neighborhood attributes related to the social (e.g., disorder, safety) and physical environment (physical deterioration, pollution); results were consistent with the authors’ premise that neighborhood conditions related with neighborhood deprivation are associated with AL [29].

### 3.8. Longitudinal Studies

Longitudinal studies (*n* = 4) were quite diverse, but essentially evaluated the influence of the neighborhood deprivation change and cumulative exposure to neighborhood deprivation. As happened for cross-sectional studies, most longitudinal studies (*n* = 3) observed a significant and positive association between neighborhood deprivation and AL. More specifically, Brody and colleagues addressed the impact of neighborhood deprivation trajectories from 11 to 19 year-old on AL and found that youth that resided in low poverty neighborhoods during childhood and moved to high poverty areas presented significantly higher AL at 19 years of age (only among those with low social support) [31]. Looking at AL at older ages, Gustafson and colleagues tested the accumulation hypothesis, i.e., if the accumulation of neighborhood disadvantages from adolescence to mid-adulthood were related to AL; analysis revealed that cumulative neighborhood disadvantage between ages 16 and 43 years was related to higher AL at age 43 years [32]. Chen et al. evaluated the skin-deep hypothesis that posits that for an impoverished population to succeed it might take a physiological toll; in this study, youth originating from more deprived neighborhoods that pursued a college degree in adolescence presented with higher AL compared to youth that did not go to college [33]. Finally, Jimenez et al., observed no significant association between neighborhood socioeconomic status and change in AL, after adjusting the models for individual socio-demographics [34].

### 3.9. Framework for Future Studies

**Figure 4:**
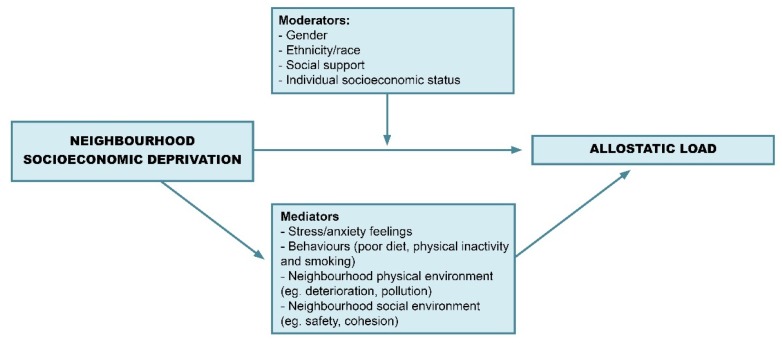
Conceptual framework for the associations between neighborhood socioeconomic deprivation and allostatic load.

Figure 4 presents a framework derived from the present scoping review that summarizes the key variables interceding the association between neighborhood socioeconomic deprivation and AL. Most of the included studies observed that neighborhood deprivation was associated with increased AL, but some relevant moderators were identified: the relation between AL and deprivation was more pronounced in certain genders, ethnic and social groups and it is also modified by social support. Furthermore, according to a few studies, the link between neighborhood socioeconomic deprivation and AL is partially mediated by detrimental behaviors, stress and anxiety feelings caused by the neighborhood conditions and by the poor social and physical environments found in impoverished areas [16,29,37].

## 4. Discussion

This scoping review has demonstrated that there is a limited number of investigations addressing the association between neighborhood socioeconomic deprivation and AL, as only 14 studies were identified. Also, the available evidence came almost exclusively from the USA and was derived from cross-sectional studies. As anticipated a wide range of measures of AL were identified and neighborhood deprivation was also measured at different geographical scales using diverse socioeconomic variables. Nevertheless, results were consistent, with 12 out of 14 studies reporting a significant association between neighborhood socioeconomic deprivation and AL after accounting for individual-level confounders.

The central issue identified in this review was the enormous heterogeneity in the biomarkers, cut-off values, control for medication intake, and mode of calculation used for the assessment of AL. Such heterogeneity makes difficult to compare the studies and draw overall conclusions, even when most of the studies show a positive and significant association between the neighborhood socioeconomic deprivation and AL. Although the concept of measuring biologic stress by an AL index is decades old, no consensus exists on which biomarkers to include in calculating AL [19], with some authors agreeing that AL measurement should include neuroendocrine and immunological biomarkers [40]. Still, the most accepted approach to measure AL is by summing the number of biomarkers, from a pool of ten, for which the subject fell into the highest risk quartile; four of these markers are the so-called primary mediators (cortisol, epinephrine, norepinephrine and dehydroepiandrosterone sulphate) and six represent secondary outcomes (systolic and diastolic blood pressure, waist-hip ratio, HDL, and total cholesterol ratio and HbA_1C_). These measures refer to the function of neuroendocrine, cardiovascular, and metabolic systems. In this scoping review, we observed none of the included studies followed this standard definition. The used cut-offs varied greatly across studies: some used quartile-based definitions as proposed by Seeman and colleagues [30,31,37] and others used clinically defined thresholds [16,26,27,28,29,34,38] or z-scores [33,35,36]. Since biological data is not easily available nor cheap to manage, most of the investigations adapted AL measurements to data availability. Despite being understandable from a practical point of view, it would be advisable to run robustness checks to ascertain the predictive value of these alternative measures and to test to what extent results and conclusions are driven by biomarker choice. Only two of the included studies evaluated the quality of the derived AL measure [26,28]. Similarly, measures about neighborhood deprivation were very different across studies and nearly half of the studies used single measures to assess the socioeconomic structure of the neighborhoods, such as households below poverty line. There are advantages in using either single or multivariate measures of socioeconomic deprivation. Single measures, such as poverty rates, are easier to calculate and to replicate through time. On the other hand, multivariable indexes of deprivation are considered to better reflect the multi-dimensional nature of deprivation in a certain context, because they include a wide range of socioeconomic variables [41,42,43].

It is also important to state that the included studies may be affected by two critical geographical biases in public health. The first is the modifiable areal unit problem (MAUP), i.e., the study results may be driven by the size of the administrative units and the scale of the study area [44,45]. Indeed, in this review, as discussed in Section 3.3, we found substantial differences in the size and type of the administrative units that were used (e.g., census tracts vs. small-area market statistics). In addition, the second is the Uncertain Geographic Context Problem [46], which refers to the effect that the spatial scope and the temporal scale (e.g., activity space, individual perception, time of residency) of the neighborhood exposure may have on the study findings [45]. Focusing on residential neighborhoods can produce a considerable amount of uncertainty in research results, given that most people spent the vast majority of their time outside the neighborhood of residence. In all the included studies, census areas were used as a proxy of the residential neighborhood, although a few accounted for some mediation variables of the perceived neighborhood environment, that may not fully match with the residential neighborhood (i.e., administrative units) that was used to measure neighborhood socioeconomic deprivation [16,29,37]. In future studies, one way to account for this issue is to use qualitative methods and mobile tracking technology, such as global positioning systems (GPS), to identify people’s true geographic and temporal contexts [47,48]. Another identified issue in the definition of the geographic context relates to the fact that most investigations disregarded spatiotemporal population dynamics and the fact that individuals are mobile agents. Only four of the included studies accounted for the fact that participants change their home address and that these residential trajectories might play an important role on the studied outcomes, more specifically AL [16,31,32,34]. Indeed, one of the studies found that youth that resided in low poverty neighborhoods during childhood and moved to high poverty areas presented significantly higher AL [31].

Most of the reviewed studies assumed a direct-contextual path, i.e., that neighborhood deprivation directly influences AL. Although this direct path may exist, it is more plausible that the association between AL and deprivation happens through multiple indirect pathways: health-related behaviors, physical factors (pollution, degradation) and social environmental stressors (unsafety, discrimination, low trust and support) present in the neighborhood of residence. Even though the results are not directly comparable and somehow counterintuitive, our review suggests that indeed unhealthy behaviors (tobacco, diet), physical and social environmental stressors explain part (but not all) of the effect of neighborhood deprivation on AL [16,29,37]. Since the presence of a relationship between neighborhood deprivation and AL is relatively established and consistent, attention should be given to the identification of the life course mediators and paths that explain why individuals in deprived neighborhood have higher AL.

Also worth mentioning is the fact that a substantial number of the retrieved studies found that the association between neighborhood socioeconomic deprivation and AL is moderated by social and demographic characteristics. Minorities, men, low SES individuals, individuals with poor social support seem to be more susceptible to the harmful effects of neighborhood deprivation. The need to address effect modification has been pointed out by several investigations dealing with downstream health outcomes. For instance, the “deprivation amplification” hypothesis states that coming from a low SES background and residing in low SES neighborhoods might exert a cumulative detrimental influence on health, so that low SES individuals living in low SES neighborhoods might carry a higher health risk [11]. Gender [49,50] and racial [51,52] differences in the neighborhood effects have also been widely reported regarding outcomes other than AL. Women and men perceive and navigate in their residential environments in different ways. Although evidence is far from being consistent, several studies suggest perceptions about the residential social and physical environment exert a stronger influence among women; but, on the other hand, men tend to be more exposed to physical stressors [49]. Suffice then to say, that future studies should explore the presence of cross-level interactions between individual and contextual determinants.

There are limitations to this review. The scoping review methodology, despite being the most adequate for our study aim, presents some drawbacks. For instance, it does not allow to meta-analyze the associations between neighborhood deprivation and AL, as no restrictions were imposed to the included studies to guarantee homogeneity. Yet, since research on this topic is new, heterogeneous, and sparse, meta-analysis would not be feasible. Additionally, as in other reviews of the literature, ours is subject to publication bias; it is recognized that studies showing significant associations are more likely to be published [53].

Even so, this review offers a comprehensive and detailed overview of the state of the literature and systematically map the main findings, methodological and analytical approaches applied in studies about the relationship between neighborhood socioeconomic deprivation and AL. This is an emerging topic in the field of social and environmental epidemiology. Subclinical signs of disease and biological dysregulation allow us to better understand the biological mechanisms by which social context affects human health and, eventually, to act before disease onset. In this review, we also highlighted the main methodological and conceptual challenges, providing orientation and a framework for future investigations, which would benefit from adopting a longitudinal and life-course approach, theoretically sound and robust measures of AL, and a more explicatory framework that investigates direct and indirect pathways.

## 5. Conclusions

To sum it up, studies evaluating the link between neighborhood socioeconomic structure and dysregulation across the body’s multiple physiological systems, AL, are new but emergent. Despite the large heterogeneity in measurements and designs, findings, so far, suggest that neighborhood socioeconomic deprivation is associated with increased AL. Such conclusions support rather solid evidence that demonstrate that neighborhood context affects a wide array of health outcomes and might help to understand how social disparities “get under the skin” to affect health and ageing.

## Figures and Tables

**Table 1:** Summary of the study findings.

Study	Design	Sample Size ^1^	Neighborhoods	Association with Neighborhood Deprivation	Physiological System ^2^	Moderators	Mediators
[36]	CS	4408	100 census tracts	YES	M, N		
[35]	CS	4410	102 census tracts	YES	M, N	G, SS	
[37]	CS	995	979 census tracts	YES			S, B
[33]	L	452	91 census tracts	YES		SES	
[34]	L	1258	318 block groups	NO			
[31]	L	420	41 census tracts	YES		SS	
[32]	L	818	374 SAMS	YES		G	
[38]	CS	550	80 focal neighborhood clusters	YES			
[29]	CS	919	69 block groups	YES			E
[30]	CS	866	55 block groups	NO			
[16]	CS	919	60 block groups	YES			E
[28]	CS	549	80 focal neighborhood clusters	YES			
[27]	CS	13,184	1805 neighborhoods	YES	M, CV		
[26]	CS	13,199	1772 census tracts	YES		R	

Design: CS (cross-sectional) and L (longitudinal); Associations: associations obtained in the final model YES (positive and significant association between neighborhood deprivation and allostatic load), NO (no significant association); Physiological system: CV (stronger associations with cardiovascular system related biomarkers); M (metabolic); and N (neuroendocrine); Moderators: G (gender), SES (individual socioeconomic status), R (race or ethnicity), SS (social support/cohesion); Mediators: B (health-related behaviors), S (stress/anxiety feelings) and E (physical or social neighborhood environment); SAMS = small-area market statistics. ^1^ Number of participants included in the study. ^2^ Physiological systems that showed a stronger association with neighborhood deprivation.

## Supplementary Materials

The following are available online at http://www.mdpi.com/1660-4601/15/6/1092/s1, Table S1: Characteristics of the studies included in the scoping review.
