# Supplementary material for: Neighborhood Socioeconomic Deprivation and Allostatic Load: A Scoping Review
Source: Int J Environ Res Public Health. 2018 May 28;15(6):1092. doi: 10.3390/ijerph15061092 (PMC6024893; doi:10.3390/ijerph15061092)
Supplement: Supplementary file 1 [file ijerph-15-01092-s001.pdf]

Table S1 – Characteristics of the studies included in the scoping review.

1st Author, year, Country and study design	Aim(s)	Sample	Neighbourhood socioeconomic deprivation measurement	Allostatic Load measurement	Control for confounding	Studied mediators	Key findings
Barber<br>2016<br>USA<br><br>Cross-sectional	(1) To examine the association between neighbourhood disadvantage and cumulative biological risk (CBR)<br>(2) To examine whether the association differs by family income and education.	**Sample**: 4408 adults from 100 census tracts<br>**Study**: Jackson Heart Study<br>**Age**: 54.5y (mean)<br>**Gender**: 63.5% female<br>**Ethnicity**: African American<br>**Region**: Metropolitan area, Jackson, Mississippi, USA.	8 variables from 2000 US Census at census tract level composed of 1200-8000 inhab/area<br>(dichotomized at the median value):<br>. % Households below poverty line<br>. % Households receiving public assistance<br>. % Occupied housing units with no vehicle<br>. % Adults ≥25 years with less than high school education<br>. % Unemployed individuals ≥16 years in the civilian labour force<br>. % Housing units unoccupied<br>. % Occupied housing units with >1 person/room (crowding)<br>. % Female-headed households	**Calculus**: Score derived from the sum of biomarker z-scores, covering: cardiovascular metabolic, inflammatory, neuroendocrine systems.<br><br>**Biomarkers**: 8 biomarkers<br>. Systolic BP<br>. Systolic BP<br>. Resting heart rate<br>. HbA1c<br>. Total cholesterol–HDL cholesterol ratio<br>. Waist circumference<br>. High sensitivity C-reactive protein<br>. Serum cortisol	. Age, gender, family income, educational attainment<br><br>. Health behaviours (physical activity, percentage of calories from dietary fat, cigarette smoking status, and alcohol consumption)	None	Association between living in a disadvantaged neighbourhood and CBR was strongest among men living in neighbourhoods with low levels of social cohesion (b=0.63, standard error=0.32)
Barber<br>2016<br>USA<br><br>Cross-sectional	(1) To examine the association between neighbourhood disadvantage and cumulative biological risk (CBR)<br>(2) To examine whether the association differs by family income and education.	**Sample**: 4410 adults from 102 census tracts<br>**Study**: Jackson Heart Study<br>**Age**: 54.5y (mean)<br>**Gender**: 63.5% female<br>**Ethnicity**: African American<br>**Region**: Metropolitan area, Jackson, Mississippi, USA.	8 variables from 2000 US Census at census tract level<br>(dichotomized at the median value):<br>. % Households below poverty line<br>. % Households receiving public assistance<br>. % Occupied housing units with no vehicle<br>. % Adults ≥25 years with less than high school education<br>. % Unemployed individuals ≥16 years in the civilian labour force<br>. % Housing units unoccupied<br>. % Occupied housing units with >1 person/room (crowding)<br>. % Female-headed households	**Calculus**: Score derived from the sum of biomarker z-scores, covering: cardiovascular metabolic, inflammatory, neuroendocrine systems.<br><br>**Biomarkers**: 8 biomarkers<br>. Systolic BP<br>. Systolic BP<br>. Resting heart rate<br>. HbA1c<br>. Total cholesterol–HDL cholesterol ratio<br>. Waist circumference<br>. High sensitivity C-reactive protein<br>. Serum cortisol	. Age, gender, family income, educational attainment<br>. Health behaviours (physical activity, percentage of calories from dietary fat, cigarette smoking status, and alcohol consumption)<br>. Anti-hypertensive and lipid-lowering medication (sensitivity analysis)	None	Living in a disadvantaged neighbourhood was associated with greater CBR after covariate adjustment (b=0.18, p<0.05).<br><br>Interactions showed a weaker association for individuals with ≤high school education but were not statistically significant.<br><br>System-specific analysis revealed stronger associations in the sub index of the metabolic and neuroendocrine system.
Robinette<br>2016<br>USA<br><br>Cross-sectional	Examine whether lower neighborhood income would relate to higher	**Sample**: 995 adults from 979 census tracts.<br>**Study**: Biomarker Project of the Midlife in the United States (MIDUS)	Neighbourhood income: median household income at the census tract level, collected from the 2000 US Census.	**Calculus**: Sum of dichotomized biomarkers based on clinically accepted "high risk" criteria according to different biological systems.	.Individual income<br>.Age<br>.Gender	.Neighbour hood safety (psychologi cal)	People living in higher income neighbourhoods have lower AL (b=-0.05, p<0.001).

	allostatic load (AL), or physiological well-being, through psychological, affective, and behavioral pathways.	**Age**: 55.2y (mean)<br>**Gender**: 55.5% females<br>**Ethnicity:** Not stated<br>**Region**: USA		**Biomarkers**: 24 biomarkers<br>. Systolic BP<br>. Diastolic BP<br>. Resting heart rate<br>. Cortisol<br>. DHEAS-S<br>. C-reactive protein<br>. Fibrinogen<br>. IL-6<br>. e-Selectin<br>. ICAM-1<br>. HbA1c<br>. Fasting glucose<br>. HOMA-IR<br>. HDL<br>. LDL<br>. Triglycerides<br>. BMI<br>. waist hip ratio<br>. low frequency spectral power<br>. high frequency spectral power<br>. standard deviation of IBIs<br>. RMSSD<br>. Epinephrine<br>. Norepinephrine		.Neighbourhood<br>.cohesion (psychological)<br>.Affective distress<br>.Health behaviours	Partially explained by affective and behavioural pathways.
Chen<br>2015<br>USA<br><br>Longitudinal	To test the skin-deep resilience hypothesis by assessing the effect of residing in low SES neighbourhoods on AL and see if it differs according to college attendance.	**Sample:** 452 youth from 91 census tracts.<br>**Study**: Strong African American Families Healthy Adult Panel (SHAPE)<br>**Age**: 20.4y (mean) in the last assessment<br>**Gender**: 55% female<br>**Ethnicity**: African American<br>**Region**: disadvantaged and rural counties of Georgia, UA.	Neighbourhood poverty (percentage of residents in a neighbourhood living below poverty) at census tract-level from 2010 US Census Bureau's American Community Survey.	**Calculus**: Scored derived sum biomarker z-scores.<br><br>**Biomarkers**: 6 biomarkers.<br>. Systolic BP<br>. Diastolic BP<br>. Urinary epinephrine<br>. Urinary norepinephrine<br>. Urinary cortisol<br>. BMI	. Age<br>. Gender<br>. Neighbourhood ethnic composition<br>. Cumulative family socioeconomic risk<br>. Primary caregivers' college attendance<br>. Co-habitation<br>. Financial stress<br>. Hours of employment	None	Among those not in college, neighbourhood poverty was not associated with AL. However, among those in college, coming from a neighbourhood with greater poverty was associated with higher AL (b=5.774, p=0.022).<br>AL was highest among youth who, at age 19, lived in high-poverty neighbourhoods and were attending college.
Jiménez<br>2015<br>USA<br><br>Longitudinal	To test the impact of baseline neighbourhood context (neighbourhood socioeconomic status and relative income of	**Sample**: 1258 adults from 318 block groups<br>**Study**: The Boston Puerto Rican Health Study<br>**Age**: 57.1y (mean)<br>**Gender**: 70% female<br>**Ethnicity**: Puerto Ricans	Score derived from 6 variables available in 2000 US Census at block-group level:<br>. % adults >25 years with less than a high school education,<br>. % male unemployment<br>. % households with income below poverty	**Calculus**: Score derived from dichotomized variables; dichotomization based on clinical cut offs when available (for neuroendocrine parameters the upper quartile was used).<br>**Biomarkers**: 11 biomarkers<br>. Systolic BP	. Gender, age and individual-level socioeconomic position<br>. City and length of time lived at the current residence<br>. Baseline AL	None	The neighbourhood deprivation score only showed significance (β =-0.120, p=0.05) when jointly adjusting for relative income.<br>After adjustment for individual-level SES and demographics the association became non-significant.

	individuals compared to their neighbours) on allostatic load two years later.	**Region**: Greater Boston area, USA; mainly urban but also suburban	. % households receiving public assistance<br>. % female-headed households with children<br>. Median household income	. Diastolic BP<br>. Waist–hip ratio<br>. Serum HDL cholesterol<br>. Total cholesterol<br>. Serum DHEA-S<br>. Plasma concentration of total glycosylated haemoglobin<br>. Urinary cortisol<br>. Urinary norepinephrine<br>. Urinary epinephrine<br>. C-reactive protein<br>. An additional point was assigned to account for relevant medication use when the parameter was within the established cut off.			
Brody<br>2014<br>USA<br><br>Longitudinal	(1) To determine the effects that changes in neighbourhood poverty levels have on AL<br>(2) To investigate the possibility that the receipt of emotional support during adolescence has a protective effect.	**Sample**: 420 youth in 41 census tracts.<br>**Age**: mean: 11.2y (first assessment); 19.2y (last).<br>**Gender**: 53% female<br>**Ethnicity**: African American<br>**Region**: disadvantaged and rural counties of Georgia, USA	Neighbourhood poverty level (proportion of households below the federal poverty level) obtained from 2000 STF3A census tract data (11y) and 2010 US Census Bureau's American Community<br>Survey (19y).	**Calculus**: Scored derived from dichotomized biomarkers; dichotomized based on quartiles (top quartile=high risk).<br><br>**Biomarkers**: 6 biomarkers.<br>. Systolic BP<br>. Diastolic BP<br>. Urinary epinephrine<br>. Urinary norepinephrine<br>. Urinary cortisol<br>. BMI	. Gender<br>. Family poverty status at age 11, age 19, and their interaction<br>. Primary caregiver employment status<br>. youths' diet, smoking, alcohol use<br>. Perceived life stress at age 19<br>. Residential mobility	None	Living in neighbourhood environments that become poorer over time was associated with AL (b=0.21, p=0.05), but only when they did not receive high levels of emotional support (b=0.61 p<0.001).{Slopen, 2012 #9;Schulz, 2013 #32;Schulz, 2012 #28;Robinette, 2016 #30;Merkin, 2009 #36;King, 2011 #58;Gustafsson, 2014 #33;Chen, 2015 #39;Brody, 2014 #35;Bird, 2010 #38;Barber, 2016 #74;Barber, 2016 #59}
Gustafsson 2014<br>Sweden<br><br>Longitudinal	(1) Examine whether socioeconomic disadvantage of the residence neighbourhood at 4 time points during the life course were cumulatively related to AL in mid-adulthood.<br>(2) Explore the cumulative effect on AL separately in women and men.	**Sample**: 818 individuals in 374 SAMS.<br>**Study**: Northern Swedish Cohort<br>**Age**: 16y (wave 1), 21y (wave 2) 30y (wave 3) and 43y (wave 4).<br>**Gender**: 47.9% female<br>**Ethnicity**: Not stated<br>**Region**: middle-sized industrial town in northern Sweden	Composite index derived from 8 variables from Statistics Sweden at SAMS level (mean 1000 individuals per area)<br>. Annual household income ≤ 10th percentile of the Swedish population<br>. Annual household income ≥ 90th percentile of the Swedish population<br>. Housing allowance<br>. Wealth tax payment<br>. Non.employment<br>. Single-parent households<br>. Occupational status<br>. Educational attainment.	**Calculus**: Score derived from dichotomized biomarkers; tertiles separately for women and men<br><br>**Biomarkers**: 12 biomarkers at 43 years:<br>. Systolic BP<br>. Diastolic BP<br>. BMI<br>. Waist circumference<br>. Fasting glucose<br>. Total cholesterol<br>. HDL cholesterol<br>. Triglycerides<br>. Apolipoprotein A1<br>. Apolipoprotein B<br>. C-reactive protein	. Gender<br>. Cumulative socioeconomic disadvantage<br>. Social and material adversity	None	The cumulative neighbourhood disadvantage between ages 16 and 43 years was related to AL at age 43 years after adjusting for personal living conditions in the total sample (b=0.11; p=0.004) and in men (b=0.16; p=0.004), but not in women (b=0.07; p=0.248).

				. Cortisol area under the curve			
Slopen<br>2014<br>USA<br><br>Cross-sectional	To evaluate the association between childhood adversity and CBR in adulthood, and to examine whether this relationship varied by adult neighbourhood affluence.	**Sample:** 550 adults in 80 focal neighbourhood clusters.<br>**Study**: Chicago Community Adult Health Study (CCAHS)<br>**Age**: 44.3y (mean)<br>**Gender**: 54.1% females<br>**Ethnicity**: Blacks, Non-Hispanic whites, Native and<br>foreign Hispanics<br>**Region**: Chicago city, USA	Neighbourhood affluence calculated using data at census tract level from 2000 US Census (average value of standardized variables):<br>. Proportion of employed civilians aged ≥16 years in professional/managerial occupations<br>. Proportion of individuals aged ≥25 years who have completed ≥16 years of education<br>. Median home values<br>(Categorized into tertiles of neighborhood affluence)	**Calculus**: Sum of dichotomized biomarkers based on clinically accepted "high risk" criteria.<br>**Biomarkers**: 8 biomarkers<br>. Systolic BP<br>. Diastolic BP<br>. Resting heart rate<br>. Glycosylated Hemoglobin A1c<br>. C-reactive protein<br>. Waist size<br>. Total cholesterol<br>. HDL cholesterol	.Gender<br>. Age<br>. Ethnicity<br>.Medication	None	Higher neighbourhood affluence was associated with a lower CBR, independent of childhood adversity and the other covariates in the models (IRR=0.82, 95% CI=0.74, 0.92).
Schulz<br>2013<br>USA.<br><br>Cross-sectional	To examine contributions of observed and perceived neighbourhood characteristics in explaining associations between neighbourhood poverty and CBR.	**Sample**: 919 adults older than 25 years from 69 block groups<br>**Study**: Healthy Environments Partnership Community Survey<br>**Age**: 46.3y (mean)<br>**Gender**: 52.3% female<br>**Ethnicity**: non-Hispanic White, non-Hispanic Black or Mexican-American<br>**Region**: Detroit, Michigan, USA.	. Neighbourhood poverty (percentage of households below the poverty line at the census block group level, derived from 2000 US Census data).	**Calculus**: Sum of dichotomized biomarkers based on clinically accepted "high risk" criteria.<br>**Biomarkers**: 8 biomarkers.<br>. Diastolic BP<br>. Systolic BP<br>. Waist circumference<br>. Glucose<br>. Total cholesterol<br>. Triglycerides<br>. HDL<br>. LDL<br>.Self-reported use of medication for hypertension, diabetes and hypercholestemia	. Individual age<br>. Gender<br>. Household income below poverty<br>. Education<br>. Race and ethnicity<br>. Metabolic<br>. Healthy eating index<br>. Smoking and alcohol use<br>. Neighbourhood percent African American and percent Latino	Perceived (social and physical environmen t) and observed (disorder, deterioratio n) neighbourh ood characteristi cs	There is an inverse association between neighbourhood poverty and CBR, with those residing in neighbourhoods with the highest levels of poverty most likely to have higher CBR.<br><br>Observed and perceived neighbourhood characteristics mediated this association.
Wallace<br>2013<br>USA<br><br>Cross-sectional	To examine the relationships between allostatic load, race, and adverse birth outcomes within the context of neighbourhood-level poverty.	**Sample**: 866 adult women from 55 census block groups.<br>**Study**: Bogalusa Heart Study<br>**Age**: mean of 13.4y and 15.8y for African American and White, respectively<br>**Gender**: 100% female<br>**Ethnicity**: 352 African American and 514 White<br>**Region**: Louisiana, USA.	Neighbourhood poverty: % of households living below the federal poverty level at block group-level (mean 600-3000 inhabit/areas) available for the 1990 and 2000 census.<br>Dichotomized in high/low poverty	**Calculus**: Score derived from dichotomized biomarkers based on age-adjusted quantiles.<br><br>**Biomarkers**: 9 biomarkers<br>. Systolic BP<br>. Diastolic BP<br>. Total cholesterol<br>. HDL cholesterol<br>. LDL cholesterol<br>. Triglycerides<br>. Glucose<br>. Insulin<br>. Waist circumference.	Propensity score matching. Adjustment for maternal age, smoking during pregnancy, year of BHS examination and years between examination and conception.	None	Twenty-two percent (n=76) of African American mothers had<br>high allostatic load and lived in high poverty neighbourhoods compared to only 3.3% (n= 17) of white women. Yet, there was no significant association between neighbourhood poverty level and allostatic load in this sample with adjustment for race (data not shown).
Schulz<br>2012<br>USA	(1)To understand whether neighbourhood	**Sample:** 919 adults from 69 block groups	. Neighbourhood poverty (percentage of households below the poverty line) at	**Calculus**: Sum of dichotomized biomarkers based on clinically accepted "high risk" criteria.	. % of Non-Hispanic Black<br>. % of Hispanic	.Psychosoci al stress associated	Neighbourhood poverty was significantly (b=0.012; p =0.019) and positively associated with AL.

Cross-sectional	poverty is associated with AL, independent of household income<br>(2)To test the hypothesis that relationships between neighbourhood poverty and AL are mediated by self-reported psychosocial stress and health related behaviours.	**Study**: Healthy Environments Partnership Community Survey<br>**Age**: 46.3y (mean)<br>**Gender**: 52.3% female<br>**Ethnicity**: non-Hispanic White, non-Hispanic Black or Mexican-American<br>**Region**: Detroit, Michigan, USA.	the census block group level, derived from 2000 US Census data.	**Biomarkers**: 8 biomarkers.<br>. Diastolic BP<br>. Systolic BP<br>. Waist circumference<br>. Glucose<br>. Total cholesterol<br>. Triglycerides<br>. HDL<br>. LDL<br>.Self-reported use of medication for hypertension, diabetes and hypercholestemia	. Individual age<br>. Gender<br>. Household income below poverty<br>. Education<br>. Race/ethnicity<br>. length of residence (sensitivity analysis)	with neighbourhood conditions (13 indicators of social and physical characteristics of the neighbourhood environment.)<br>. Health behaviours	The relationship was mediated by neighbourhood environment stress.<br>No differential effect according to race.
King<br>2011<br>USA<br><br>Cross-sectional	To examine the role of neighbourhood context in CBR and racial/ethnic and socioeconomic disparities.	**Sample:** 549 adults in 80 focal neighbourhood clusters.<br>**Study**: Chicago Community Adult Health Study (CCAHS)<br>**Age**: 43y (mean)<br>**Gender**: 53.9% female<br>**Ethnicity**: Blacks, Non-Hispanic whites, Native and foreign Hispanics<br>**Region**: Chicago city, USA.	Neighbourhood socioeconomic disadvantage:<br>. Proportion of households with incomes of less than 15000USD and of at least 50000USD<br>. Families in poverty<br>. Households on public assistance<br>. Unemployment<br>. Vacant housing units<br><br>Neighbourhood affluence:<br>. Employed civilians;<br>. Individuals who have completed 16 or more years of education;<br>. Median home values.	**Calculus**: Sum of dichotomized biomarkers based on clinically accepted "high risk" criteria.<br><br>**Biomarkers**: 8 biomarkers<br>. Systolic BP<br>. Diastolic BP<br>. Resting heart rate<br>. Glycosylated Hemoglobin A1c<br>. C-reactive protein<br>. Waist size<br>. Total cholesterol<br>. HDL cholesterol	. Race/ethnicity<br>. Gender<br>. Age<br>. Immigrant generation<br>. Educational attainment<br>. Income<br>. Health behaviours (physical activity, fruit and vegetable intake, smoking and drinking alcohol)	None	Neighbourhood affluence predicted lower levels of CBR (IRR=0.82, p<0.027), but neighbourhood disadvantage was not significantly associated with CBR (IRR=1.00, p<0.948)
Bird<br>2010<br>USA<br><br>Cross-sectional	To assess whether neighbourhood socioeconomic status is independently associated with disparities in AL.	**Sample**: 13,184 adults from 1805 neighbourhoods<br>**Study**: Third National Health and Nutrition Examination Survey (1988-1994) (NHANES III)<br>**Age**: 45y (mean)<br>**Gender**: 51.9% female<br>**Ethnicity**: non-Hispanic White, non-Hispanic Black or Mexican-American.<br>**Region**: USA	Sum of six census tract-level standardized variables:<br>. % adults ≥25 years with less than a high school education<br>. % male unemployment<br>. % households with income below the poverty line<br>. % households receiving public assistance<br>. % female-headed households with children<br>. median household income	Calculus: Sum of dichotomized biomarkers based on clinically accepted "high risk" criteria.<br><br>Biomarkers: 9 biomarkers pertaining to inflammatory, metabolic and cardiovascular system.<br>. Albumin (inflammatory)<br>. C-reactive protein (inflammatory)<br>. Waist-to-hip ratio (metabolic)<br>. Total cholesterol (metabolic)<br>. HDL cholesterol (metabolic)	. Age<br>. Gender<br>. Race/ethnicity<br>. Education<br>. Poverty Income Ratio<br>. Marital status<br>. Nativity	None	Living in a lower SES neighbourhood was associated with worse AL (AL difference =−0.046; 95%CI[1] −0.079, −0.012).<br><br>The relationship between neighbourhood deprivation and AL did not vary significantly by gender, household poverty, or race/ethnicity.<br><br>System-specific analysis revealed stronger associations in the sub index of the metabolic and cardiovascular system.

				. Glycated haemoglobin (metabolic)<br>. Systolic BP (cardiovascular)<br>. Diastolic BP (cardiovascular)<br>. Resting heart rate (cardiovascular)			
Merkin<br>2009<br>USA<br><br>Cross-sectional	To evaluate the association between neighbourhood SES and AL separately in three major race/ethnic groups.	**Sample:** 13,199 adults from 1772 census tracts<br>**Study:** Third National Health and Nutrition Examination Survey (1988-1994) (NHANES III).<br>**Age:** 41y (median)<br>**Gender**: 51.9% females<br>**Ethnicity**: non-Hispanic whites, non-Hispanic blacks and Mexican Americans.<br>**Region**: USA	Sum of six census tract-level standardized variables (then categorized in quintiles):<br>. % of family households with children not headed by female<br>. % of male population ages 16 and older that is employed<br>. % of households that do not receive public assistance income<br>. % of households with income higher than the poverty threshold<br>. Median household income<br>. % of population 25 and older with high school diploma or higher education	**Calculus**: Sum of dichotomized biomarkers based on clinically accepted "high risk" criteria.<br><br>**Biomarkers**: 9 biomarkers On inflammatory, metabolic and cardiovascular systems:<br>. Albumin<br>. C-reactive protein<br>. Waist-to-hip ratio<br>. Total cholesterol<br>. HDL cholesterol<br>. Glycated haemoglobin<br>. Systolic BP<br>. Diastolic BP<br>. Resting heart rate	.Age<br>.US birth<br>.urban/rural location<br>.individual SES (education and poverty income ratio)	None	Blacks living in the lowest SES neighbourhoods had over twice the odds of a high AL score as compared with those living in the highest neighbourhood SES quintile (OR: 2.2, 95%CI: 1.1, 4.1).<br><br>Non-significant trends were seen for both whites and Mexican Americans.

AL=Allostatic load; CBR=Cumulative biological risk; BMI=Body mass index; BP=Blood Pressure; DHEA-S=Dehydroepiandrosterone sulfate; HbA1C=glycosylated hemoglobin; HDL=High-density lipoprotein; HOMA-IR=Homeostatic model assessment insulin resistance; IBIs=Inter-beat intervals; ICAM-1=Intercellular adhesion molecule 1; IL-6= Interleukin 6; IRR=Incidence Rate Ratio; LDL=Low-density lipoprotein; OR=Odds ratio; RMSSD=Root Mean Square of the Successive Differences (Heart rate variability); SAMS= small-area market statistics; SES=Socioeconomic status; 95%CI=95% Confidence Interval.
